# 

**Published:** 1830-03

**Authors:** 


### MONTHLY LIST OF MEDICAL BOOKS.

[*Medical Works cannot be entered on this List except a copy be sent for the purpose; the titles of Books having frequently been transmitted to us, as published, which have not appeared for weeks, or even months, after.*]

A Popular Summary of Vaccination, with Reference to its Efficacy, and probable Causes of Failure; as suggested by extensive Practical Experience. By JOHN MARSHALL, Esq. Member of the Royal College of Surgeons, and District Vaccinator to the National Vaccine Establishment.—8vo. pp. 95. Underwood, London, 1830.

Introductory Lectures to a Course of Military Surgery, delivered in the University of Edinburgh. By GEORGE BALLINGALL, M D. F.R.S.E. Regius Professor of Military Surgery, &c.—8vo. pp. 246. Black, Edinburgh; Longman, London, 1830.

Illustrations of some of the principal Diseases of the Ovaria, their Symptoms and Treatment; to which are prefixed, Observations on the Structure and Functions of those Parts in the Human Being and in Animals. By EDWARD J. SEYMOUR, M.D. Fellow of the Royal College of Physicians of London, and one of the Physicians to St. George's Hospital. With fourteen Lithographic Engravings.—8vo. pp. 126. Longman, 1830.

We shall give an analysis of this very important contribution to Pathology in our next Number.

